# Modulation of Gut Microbiota by *Lonicera caerulea* L. Berry Polyphenols in a Mouse Model of Fatty Liver Induced by High Fat Diet

**DOI:** 10.3390/molecules23123213

**Published:** 2018-12-05

**Authors:** Shusong Wu, Ruizhi Hu, Hironobu Nakano, Keyu Chen, Ming Liu, Xi He, Hongfu Zhang, Jianhua He, De-Xing Hou

**Affiliations:** 1 Core Research Program 1515, Hunan Collaborative Innovation Center for Utilization of Botanical Functional Ingredients, Hunan Agricultural University, Changsha 410128, China; wush688@126.com (S.W.); rzhi479@163.com (R.H.); liuming@hongkebio.cn (M.L.); hexi111@126.com (X.H.); 2 The State Key Laboratory of Animal Nutrition, China Agricultural University, Beijing 100193, China; zhanghongfu@caas.cn; 3 The United Graduate School of Agricultural Sciences, Kagoshima University, Kagoshima 890-0065, Japan; k8108910@kadai.jp (H.N.); k4164345@kadai.jp (K.C.)

**Keywords:** polyphenols, *Lonicera caerulea* L., fatty liver disease, inflammation, gut microbiota

## Abstract

Polyphenols from the *Lonicera caerulea* L. berry have shown protective effects on experimental non-alcoholic fatty liver disease (NAFLD) in our previous studies. As endotoxins from gut bacteria are considered to be the major trigger of inflammation in NAFLD, this study aims to clarify the regulatory effects of *L. caerulea* L. berry polyphenols (LCBP) on gut microbiota in a high fat diet (HFD)-induced mouse model. C57BL/6N mice were fed with a normal diet, HFD, or HFD containing 0.5–1% of LCBP for 45 days. The results revealed that supplementation with LCBP decreased significantly the levels of IL-2, IL-6, MCP-1, and TNF-α in serum, as well as endotoxin levels in both serum and liver in HFD-fed mice. Fecal microbiota characterization by high throughput 16S rRNA gene sequencing revealed that a HFD increased the *Firmicutes*/*Bacteroidetes* ratio, and LCBP reduced this ratio by increasing the relative abundance of *Bacteroides,* *Parabacteroides*, and another two undefined bacterial genera belonging to the order of *Bacteroidales* and family of *Rikenellaceae*, and also by decreasing the relative abundance of six bacterial genera belonging to the phylum *Firmicutes*, including *Staphylococcus*, *Lactobacillus*, *Ruminococcus*, and *Oscillospira*. These data demonstrated that LCBP potentially attenuated inflammation in NAFLD through modulation of gut microbiota, especially the ratio of *Firmicutes* to *Bacteroidetes*.

## 1. Introduction

Phytochemicals present in functional foods, such as cool climate berries, offer a great hope as an alternative therapy for chronic disorders [1]. *Lonicera caerulea* L., also known as blue honeysuckle, honeyberry or haskap, is a member of the *Caprifoliaceae* family that grows naturally in cool temperate regions in the Northern Hemisphere, such as Siberia in Russia, Hokkaido in Japan, and northern China, and has been cultivated as a healthy berry [2]. The berry of *L. caerulea* L. is rich in polyphenols, particularly anthocyanins (mainly cyanidin 3-glucoside (C3G) [3]), and possesses multiple biological functions [4]. In our previous studies, *L. caerulea* L. berry polyphenols (LCBP) have been shown to be able to protect mice against high fat diet-induced non-alcoholic fatty liver disease (NAFLD), by enhancing the antioxidant capability and inhibiting the production of inflammatory cytokines of mice [5,6]. Recent studies have suggested that endotoxins or alcohols produced by gut microbiota potentially promote the progression of NAFLD [7,8], and polyphenols may exert biological functions through interaction with gut microbiota [9]. Thus, we hypothesized that polyphenols from *Lonicera caerulea* L. berries attenuate NAFLD by regulating the intestinal bacterial community.

As a common liver disease, NAFLD has been a non-negligible threat to human health worldwide, with prevalence estimates ranging from 25% to 45% in most studies [10]. Although pathogenesis of NAFLD is complicated, factors such as high energy intake, insulin resistance, and metabolic syndrome are reported to be closely related to it [11]. Normal weight people may also develop NAFLD, although obese people have a higher risk. The presence of hepatic inflammation is considered as the most important determinant of outcome, because it promotes fibrosis in liver and leads NAFLD to the progressive non-alcoholic steatohepatitis (NASH), which can increase the risk of cirrhosis [10]. Therefore, inflammatory cytokines, triggered by endotoxins/lipopolysaccharides of intestinal bacteria [7,12], are considered to be important mediators and potential biomarkers in the pathogenesis of NAFLD [13].

Based on the information about the potential pathogenesis of NAFLD, and our previous results concerning the functions of LCBP, the present study is designed to clarify the regulatory effects of LCBP on inflammatory cytokines, endotoxin production, and correlated gut microbiota in a mouse model.

## 2. Results

### 2.1. Regulatory Effects of LCBP on Inflammatory Cytokines

**Figure 1:**
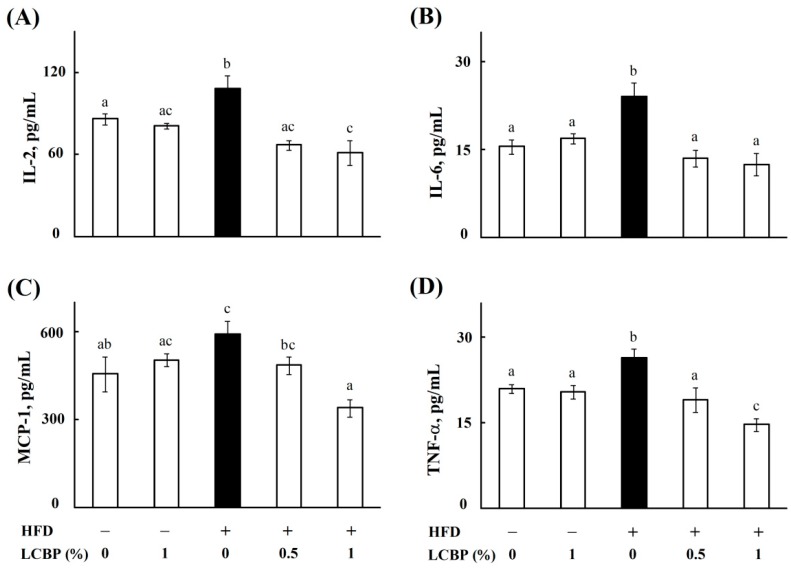
The effect of LCBP on serum levels of cytokine. IL-2 (**A**), IL-6 (**B**), MCP-1 (**C**), and TNF-α (**D**) were measured by using a Bio-Plex Pro Mouse Cytokine 23-Plex Panel kit (Bio-Rad Laboratories, Inc., Hercules, CA, USA). The data represent mean ± SD of four mice. Bars with different letters differ significantly (*p* < 0.05). HFD, High fat diet; LCBP, *Lonicera caerulea* L. berry polyphenols; IL, interleukin; MCP-1, monocytes chemotactic protein-1; TNF-α, tumor necrosis factor-alpha.

Our previous study showed that LCBP improved hepatic steatosis and insulin resistance [6], which is supposed to be mediated by cytokines [14]. Thus, a total number of 23 kinds of cytokines in mouse serum were simultaneously measured by multiplex technology, to understand the effects of LCBP on the cytokine network in this study. As shown in Figure 1, serum levels of IL-2 (A), IL-6 (B), MCP-1 (C), and TNF-α (D) were significantly increased (*P* < 0.05) in high fat diet (HFD)-fed mice, compared with normal diet (ND)-fed mice, while the amounts of other cytokines were not changed (data not shown). Supplementation with 0.5–1% of LCBP decreased (*P* < 0.05) the four cytokines to normal control level, especially serum levels of IL-2 (Figure 1A) and TNF-α (Figure 1D) in mice fed with a HFD containing 1% of LCBP, which were even lower than (*P* < 0.05) that in ND-fed mice. However, there was no significance between serum levels of cytokines in mice fed with ND or ND containing 1% of LCBP.

### 2.2. LCBP Decreased Endotoxin Level in Bboth Serum and Liver

**Figure 2:**
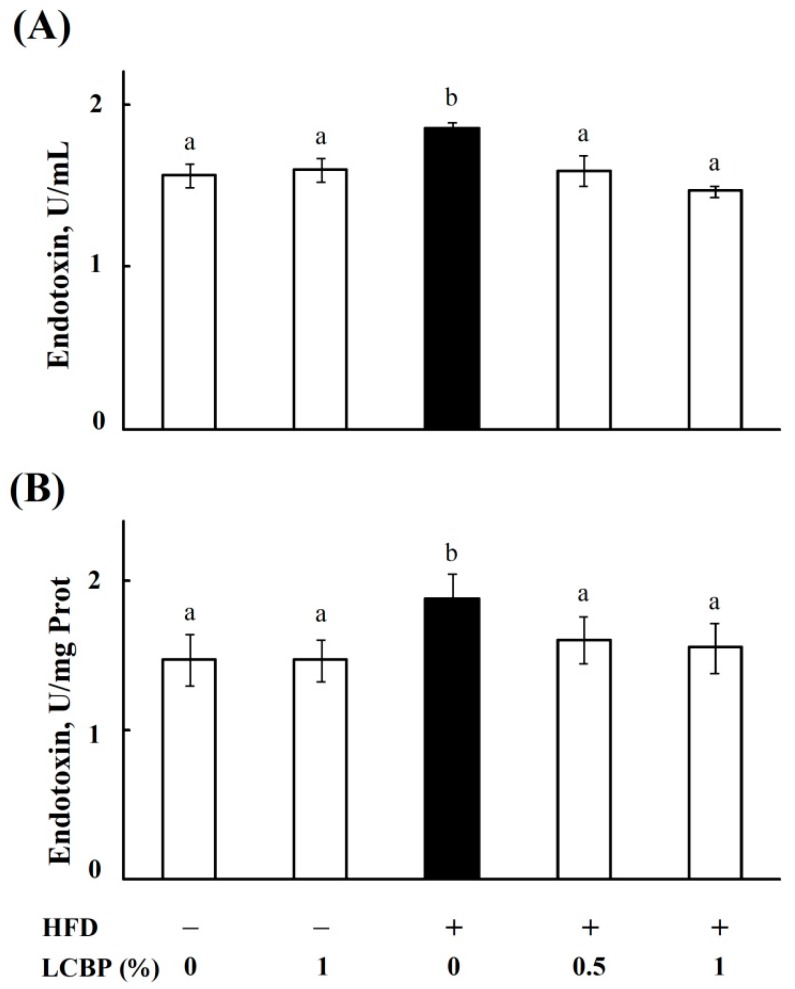
The effect of LCBP on endotoxin levels in serum and liver. The level of endotoxin in mouse serum (**A**) and liver (**B**) was measured by using a Pierce LAL Chromogenic Endotoxin Quantitation Kit (Thermo Fisher Scientific Inc., Rockford, IL, USA). The data represents the mean ± SD of four mice. Bars with different letters differ significantly (*p* < 0.05). HFD, high fat diet; LCBP, *Lonicera caerulea* L. berry polyphenols.

As endotoxins from gut bacteria might be the trigger of inflammation in NAFLD [7], we next measured the levels of endotoxins in serum and liver homogenates of mice. As shown in Figure 2, a HFD increased significantly endotoxin levels in both serum and liver, whereas the levels of endotoxins were decreased (*p* < 0.05) by supplementing with 0.5–1% of LCBP. No significant difference was observed between the ND and ND + 1% LCBP groups.

### 2.3. Modulation of Gut Microbiota by LCBP

To further understand the effects of LCBP on gut bacteria, the composition and relative abundance of microbiota were determined by using high throughput 16 rRNA gene sequencing. The diversity of fecal microbiota is shown in Table 1, HFD increased observed species richness, and Shannon diversity, compared with the ND group. Supplementation with LCBP in a HFD decreased the diversity of fecal microbiota, whereas supplementation with a ND increased the diversity.

**Figure 3:**
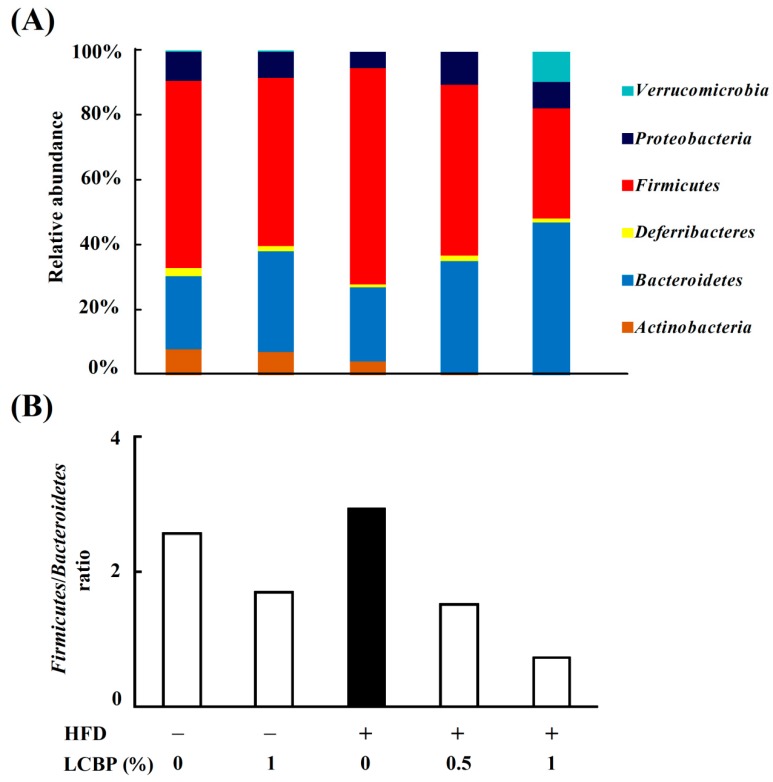
Modulation of gut microbiota by LCBP at the phylum level. Fecal microbiota were characterized by 16S rRNA gene sequencing. (**A**) The relative abundance of bacteria including *Verrucomicrobia*, *Proteobacteria*, *Firmicutes*, *Deferribacteres, Bacteroidetes* and *Actinobacteria*, at the phylum level. (**B**) The ratio of *Firmicutes* to *Bacteroidetes*, based on their relative abundance. HFD, high fat diet; LCBP, *Lonicera caerulea* L. berry polyphenols.

The relative abundance of bacteria at the phylum level showed that, compared with the ND group, the abundance of *Firmicutes* was increased in the HFD group, while the abundance of *Proteobacteria*, *Deferribacteres*, and *Actinobacteria* were decreased (Figure 3A). However, supplementation with LCBP dose-dependently decreased the abundance of *Firmicutes* and increased the abundance of *Bacteroidetes*, while the abundance of *Proteobacteria* was also increased. *Verrucomicrobia* (mostly *Akkermansia*, as shown in Figure S2) was increased only in the HFD + 1% LCBP group. The ratio of *Firmicutes* to *Bacteroidetes* was increased in the HFD group, but dose-dependently decreased by supplementing with 0.5–1% of LCBP (Figure 3B).

**Figure 4:**
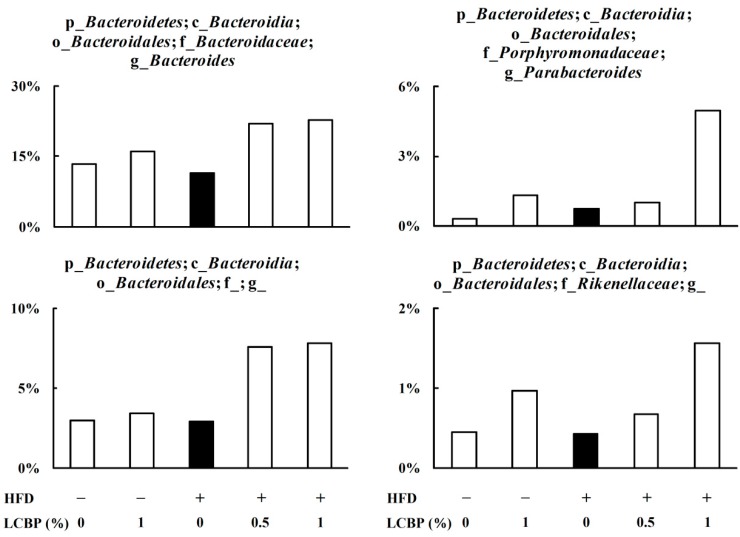
The effect of LCBP on the bacterial genera belonging to *Bacteroidetes*. Fecal microbiota were characterized by 16S rRNA gene sequencing, and the data represents the relative abundance of each bacterial genus. p_, c_, o_, f_, or g_ represents phylum, class, order, family, and genus, respectively, and a blank after the letter means undefined. HFD, high fat diet; LCBP, *Lonicera caerulea* L. berry polyphenols.

Further analysis at genus level revealed that supplementation with LCBP increased the relative abundance of genera belonging to the phylum of *Bacteroidetes* (Figure 4), including *Bacteroides*, belonging to the family of *Bacteroidaceae*, order of *Bacteroidales*, class of *Bacteroidia*; and *Parabacteroides*, belonging to the family of *Porphyromonadaceae,* order of *Bacteroidales,* class of *Bacteroidia*, as well as another two undefined genera, belonging to the order of *Bacteroidales*, family of *Rikenellaceae*, order of *Bacteroidales*, class of *Bacteroidia*.

**Figure 5:**
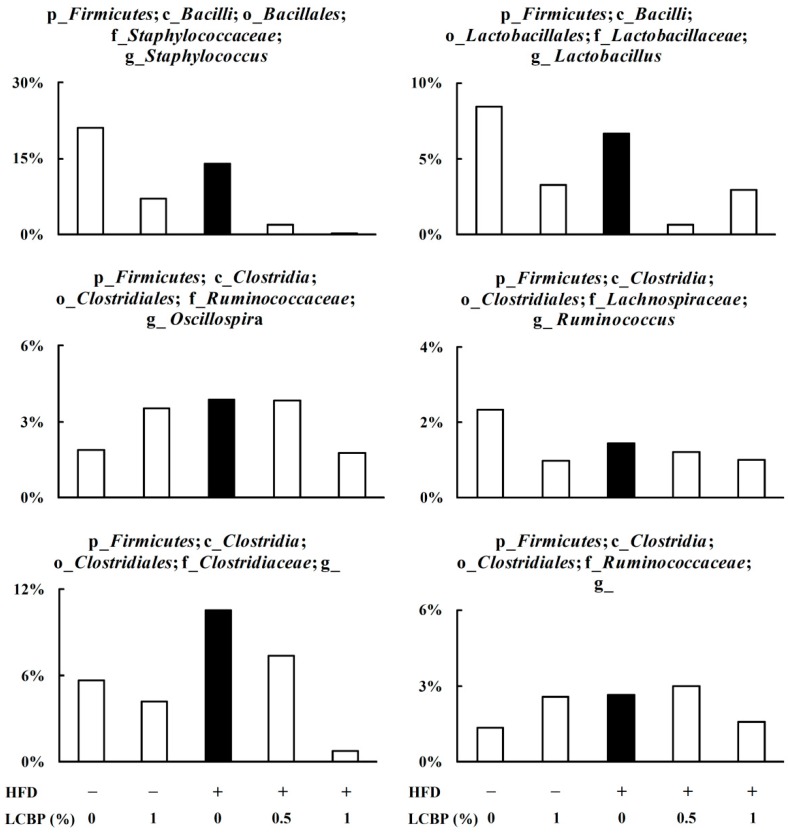
The effect of LCBP on the relative abundance of bacterial genera belonging to *Firmicutes*. Fecal microbiota were characterized by 16S rRNA gene sequencing, and the data represents the relative abundance of each bacterial genus. p_, c_, o_, f_or g_ represents phylum, class, order, family, and genus, respectively, and a blank after the letter means undefined. HFD, High fat diet; LCBP, *Lonicera caerulea* L. berry polyphenols.

However, LCBP decreased the relative abundance of the p_*Firmicutes* (Figure 5), including *Staphylococcus*, belonging to the family of *Staphylococcaceae*, order of *Bacillale*, class of *Bacilli*; *Lactobacillus*, belonging to the family of *Lactobacillacea*, order of *Lactobacillales*, class of *Bacilli*; *Oscillospira*, belonging to the family of *Ruminococcaceae*, order of *Clostridiales*, class of *Clostridia*; *Ruminococcus*, belonging to the family of *Lachnospiraceae*, order of *Clostridiales*, class of *Clostridia*; and another two undefined genera belonging to the family of *Clostridiaceae*, order of *Clostridiales*, class of *Clostridia*; and family of *Ruminococcaceae*, order of *Clostridiales*, class of *Clostridia*.

## 3. Discussion

The gut microbiota have drawn much attention in metabolic diseases recently, since gut dysbiosis has close ties to the metabolic syndrome [15], obesity [16], diabetes [17], and cardiovascular diseases [18]. Animal studies have suggested that gut microbiota might be an important player in the pathogenesis of NAFLD [19]. Characterization of fecal microbiota in NAFLD patients also revealed that the severity of NAFLD associates with gut dysbiosis and a shift in the metabolic function of the gut microbiota [20]. Gut-derived inflammation may play an important role in the onset and progression of NAFLD, and actually around 40% of inflammatory bowel disease patients also suffer from NAFLD [21]. The dysbiosis of gut microbiota may damage the intestinal epithelial barrier, and increase the intestinal permeability, which gives a way for bacteria and endotoxins to penetrate through the gut-vascular barrier, and thus promotes systemic and hepatic inflammation [22]. In the present study, HFD caused an increase in the level of endotoxins in both serum and liver of mouse, and enhanced serum levels of inflammatory cytokines including IL-2, IL-6, TNF-α, and MCP-1, which suggesting that a HFD potentially caused the dysbiosis of gut microbiota, and IL-2, IL-6, TNF-α, and MCP-1 were the potential primary response inflammatory cytokines in NAFLD. Supplementation of LCBP in the diet reduced endotoxin levels, with decreased levels of cytokines. The results indicated that LCBP might affect gut bacteria to re-establish the micro-ecological balance, as other studies also suggested, polyphenols potentially exert function through their interaction with gut microbiota [9].

Multiple species of bacteria have been reported to be closely associated with NAFLD. Characterization of gut microbiota in human stool samples revealed that a significant decrease in *Bacteroidetes* at the phylum level was found in NAFLD patients, compared with healthy controls [7,23], while the relative abundance of *Actinobacteria* [23] was increased. A recent study revealed that obese people have a higher *Firmicutes*/*Bacteroidetes* ratio, compared to normal weight people in the adult Ukrainian population [24]. In this study, the HFD increased the abundance of *Firmicutes*, but *Bacteroidetes* did not change. Supplementation of LCBP in a normal diet (ND) or HFD dose-dependently increased the relative abundance of *Bacteroidetes*, and decreased the abundance of *Firmicutes*, and, in this way, LCBP decreased the *Firmicutes*/*Bacteroidetes* ratio in a dose-dependent manner. At the genus level, *Bacteroides* and *Parabacteroides* were identified as the up-regulated bacterias by LCBP, while other two bacterial genera belong to the order of *Bacteroidales* and family of *Rikenellaceae* were also increased. Previous studies demonstrated that the relative abundance of *Bacteroides* and *Parabacteroides* were decreased in the gut of NAFLD patients [23], or HFD-induced rats [25]. However, the abundance of *Bacteroides* and *Parabacteroides* between ND and HFD groups had little change in this study. On the other hand, six genera belong to the phylum of *Firmicutes*, including *Staphylococcus*, *Lactobacillus*, *Ruminococcus*, and *Oscillospira* were decreased by LCBP. Recent studies suggested that the abundance of *Lactobacillus*, *Ruminococcus*, and *Oscillospira* were increased with age and associated with intestinal permeability, systemic inflammation, and macrophage dysfunction [26,27]. This may explain the increase in the levels of endotoxin and inflammatory cytokines in circulation. It is also noteworthy that the abundance of *Akkermansia*, bacteria that can prevent the development of obesity and associated complications [28,29], was increased in the HFD + 1% LCBP group, although rarely found in other groups. This will be further studied in future works.

In conclusion, supplementation with 0.5–1% of LCBP in diet decreased significantly the levels of inflammatory cytokines, including IL-2, IL-6, MCP-1, and TNF-α, in serum, as well as endotoxin levels in both serum and liver in a mice model of NAFLD. Fecal microbiota characterization by high throughput 16S rRNA gene sequencing demonstrated that LCBP reduced the *Firmicutes*/*Bacteroidetes* ratio, which was increased by a HFD. These data provided a scientific basis for understanding the pathogenesis of NAFLD, and the preventive effects of NAFLD by intervention of polyphenols from the *Lonicera caerulea* L. berry.

## 4. Materials and Methods

### 4.1. Chemicals and Reagents 

Choline chloride, lard oil, and methionine were purchased from Sigma-Aldrich (Tokyo, Japan). Casein, cellulose, corn starch, mineral mix, sucrose, and vitamin mix were purchased from Oriental Yeast Co., Ltd., (Tokyo, Japan). LCBP were extracted as described previously [3]. Briefly, the *Lonicera caerulea* L. berries, from the Jilin province of China, were homogenized in 75% aqueous ethanol (250 g/L) for 60 min, and then filtered under reduced pressure. The filtrates were purified on a column packed with nonionic polystyrene-divinylbenzene resin (D101, Shanghai Mosu Science Equipment Co., Ltd, Shanghai, China), and then freeze-dried into powder. The retention profiles of HPLC analysis showed that C3G (59.5%) and -(-)epicatechin (EC) (25.5%) were the major phenolic components at 280 nm, and other minor anthocyanins, including cyanidin 3-rutinoside (1.8%), cyanidin 3,5-diglucoside (1.3%), peonidin 3-glucoside (7.2%), peonidin 3-rutinoside (1.9%), and pelargonidin 3-glucoside (2.3%) were also detected at 520 nm. The quantitative analysis by HPLC (Figure S1), using standards, indicated that each milligram of LCBP contains 0.37 mg C3G and 0.23 mg EC.

### 4.2. Mouse Model of NAFLD

The animal experiment was conducted according to the guidelines of the Animal Care and Use Committee of Kagoshima University (Permission No. A12005), Japan. Twenty C57BL/6N mice (male, 5 weeks of age) were purchased from SLC Inc. (Shizuoka, Japan), and housed separately in cages with wood shaving bedding under controlled temperature (25 °C) and light (12 h light/day), and had free access to feed and water. After being accommodated for 1 week, the mice were randomly divided into 5 groups (*n* = 4): A normal diet (ND) group, a ND + 1% LCBP group, a high fat diet (HFD) group, a HFD + 0.5% LCBP group, and a HFD + 1% LCBP group. Mice in different groups were fed the corresponding diets (as described in Table S1) for 45 days.

### 4.3. Measurement of Cytokines

Blood sera of mice were obtained by centrifuging at 1500× *g* for 10 min after coagulation at the end of the experiment, and serum levels of cytokines were measured by multiplex technology, as described previously [30]. In brief, cytokines, including interleukin (IL)-1α, IL-1β, IL-2, IL-3, IL-4, IL-5, IL-6, IL-9, IL-10, IL-12(p40), IL-12(p70), IL-13, IL-17, eotaxin, granulocyte colony-stimulating factor (G-CSF), granulocyte-macrophage colony-stimulating factor (GM-CSF), interferon-gamma (IFN-γ), keratinocyte-derived cytokine (KC), monocyte chemotactic protein-1 (MCP-1), macrophage inflammatory protein (MIP)-1α, MIP-1β, RANTES, and tumor necrosis factor-alpha (TNF-α), were measured with a Bio-Plex Pro Mouse Cytokine 23-Plex Panel kit (Bio-Rad Hercules, Hercules, CA, USA) by using a Bio-Plex 200 System (Bio-Rad Hercules, Hercules, CA, USA) according to the manufacturer’s instruction.

### 4.4. Measurement of Endotoxins

To measure the levels of toxins in the liver, equal amounts of liver tissues were homogenized in normal saline (0.1 g/mL) with a Speed-Mill PLUS homogenizer (Analytik Jena, Jena, Germany), and the supernatants were obtained by being centrifuged at 3500× *g* for 10 min. The levels of endotoxins in mouse serum and liver homogenates were measured by using a Pierce LAL Chromogenic Endotoxin Quantitation Kit (Thermo Fisher Scientific Inc., Rockford, IL, USA), according to the manufacturer’s manual.

### 4.5. Characterization of Gut Microbiota by 16S rRNA Gene Sequencing

Feces samples of each mouse were collected for analyzing the composition of gut bacterial communities, at the end of experiment. Briefly, the feces of each mouse were collected separately into a 1.5 mL tube, and freeze-dried to achieve constant weight. An equal amount of feces from each group was washed by DNase-free water to clean the surface, and then fecal DNA was extracted using a QIAamp PowerFecal DNA Kit (QIAGEN, Venlo, Netherlands), following the manufacturer’s manual. The V3–V4 region of the 16S rRNA gene was amplified using forward primer 341 F (5′-ACACTCTTTCCCTACACGACGCTCTTCCGATCT-NNNNN-CCTACGGGNGGCWGCAG-3′) and reverse primer 805 R (5′-GTGACTGGAGTTCA GACGTGTGCTCTTCCGATCT-NNNNN-GACTACHVGGGTATCTAATCC-3′). PCR cycling conditions consisted of an initial denaturation of 2 min at 94 °C, and 25 cycles of 30 s at 94 °C, 30 s at 55 °C, and 30 s at 72 °C. The quality of the amplified 16S rRNA gene was checked on 0.8% agarose gels. The PCR products were amplified in a second PCR employing index primer (F: 5′-AATGATACGGCGACCACCGAGATCTACAC-Index2-ACACTCTTTCCCTACACGACGC-3′; R: 5′-CAAGCAGAAGACGGCATACGAGAT-Index1-GTGACTGGAGTTCAGACGTGTG-3′). This PCR was run for 2 min at 94 °C, followed by 10 cycles of 30 s at 94 °C, 30 s at 60 °C, and 30 s at 72 °C. Amplicon sequencing was performed on the Illumina MiSeq System at Bioengineering Lab. Co., Ltd. (Atsugi, Kanagawa, Japan). 

### 4.6. Statistical Analysis

Results are expressed as means ± SD. Significant differences between groups were determined using one-way analysis of variance (ANOVA) tests, followed by Duncan’s Multiple Range test (SPSS19, IBM Corp., Armonk, NY, USA). A probability of *p* < 0.05 was considered significant.

## Figures and Tables

**Table 1:** The effect of LCBP on the diversity of gut microbiota.

Description	PD_whole_tree	Chao1	Observed_species	Shannon
ND	21.62	812.70	381.30	4.87
ND + 1% LCBP	25.58	1135.23	515.60	5.75
HFD	24.84	1377.65	486.00	5.38
HFD + 0.5% LCBP	22.79	1216.37	466.90	5.11
HFD + 1% LCBP	21.17	1015.80	398.00	5.01

**Notes.** Phylogenetic diversity (PD)**_**whole**_**tree, Chao1, and Observed species are species richness indices, and Shannon index reflects the diversity of gut microbiota. ND, normal diet group; HFD, high fat diet group; LCBP, *Lonicera caerulea* L. berry polyphenols.

## Supplementary Materials

The Supplementary Materials are available online.
