# Supplementary material for: Modulation of Gut Microbiota by Lonicera caerulea L. Berry Polyphenols in a Mouse Model of Fatty Liver Induced by High Fat Diet
Source: Molecules. 2018 Dec 5;23(12):3213. doi: 10.3390/molecules23123213 (PMC6321169; doi:10.3390/molecules23123213)
Supplement: Supplementary file 1 [file molecules-23-03213-s001.zip › molecules-389174-SI.pdf]

Supplementary Materials: The following are available online. Table S1: Dietary composition of each group; Figure S1: HPLC profiles of phenolic components in LCBP; Figure S2: The effect of LCBP on the relative abundance of *Akkermansia*. ND, normal diet; HFD, high fat diet; LCBP, *Lonicera caerulea* L. berry polyphenols.

Table S1. Dietary composition of each group.

**Components (%)**	**ND**	**ND + 1% LCBP**	**HFD**	**HFD + 0.5% LCBP**	**HFD + 1% LCBP**
Lard	6	6	40	40	40
Casein	21	21	21	21	21
Sucrose	10	10	10	10	10
Cellulose	4	4	4	4	4
Mineral mix	3.5	3.5	3.5	3.5	3.5
Vitamin mix	1	1	1	1	1
Choline chloride	0.2	0.2	0.2	0.2	0.2
Methionine	0.3	0.3	0.3	0.3	0.3
Corn starch	54	53	20	19.5	19
LCBP	0	1	0	0.5	1
Total	100	100	100	100	100
Calories (kcal/100g)	377.94	374.13	555.08	553.18	551.27

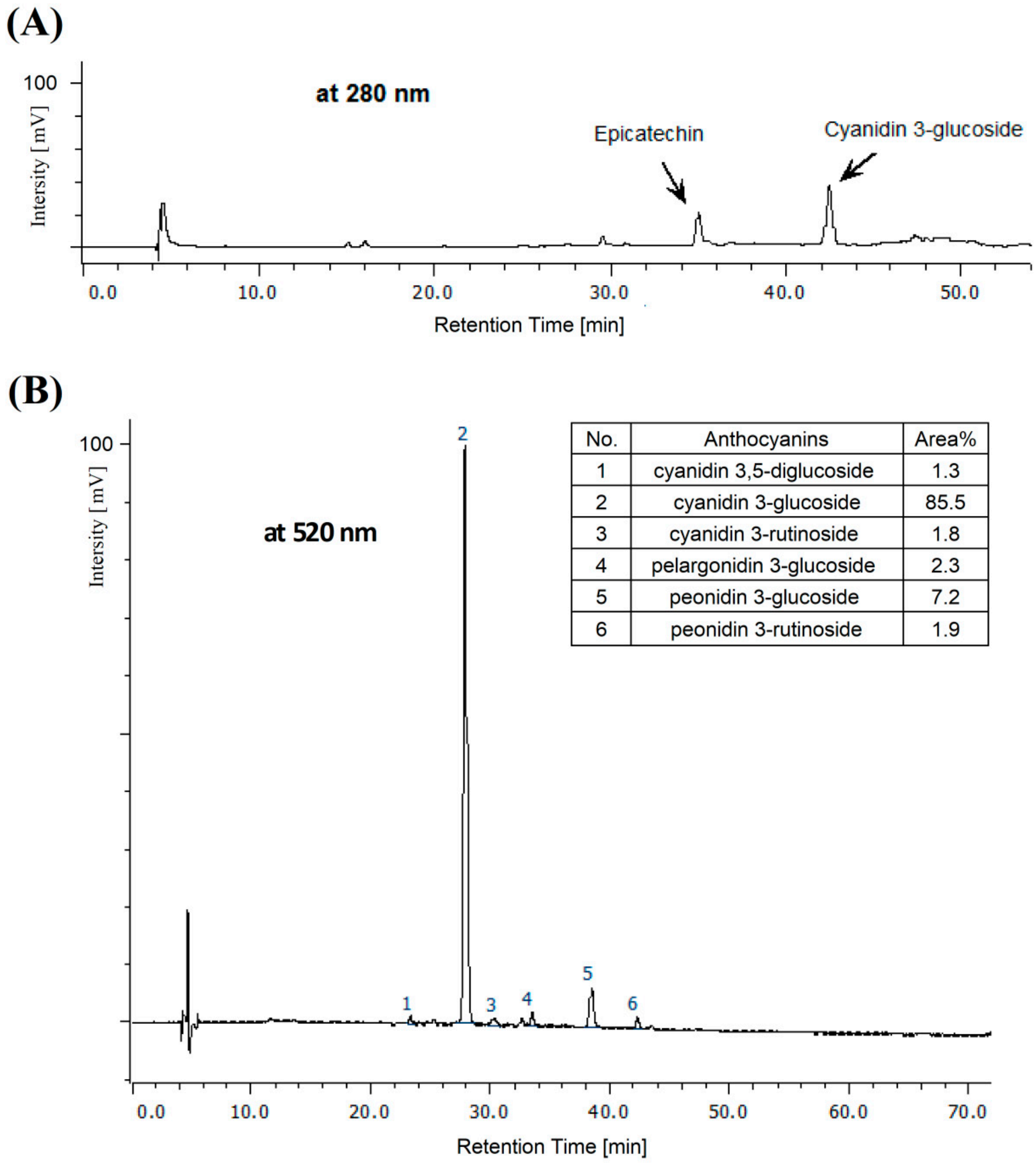

No.	Anthocyanins	Area%
1	cyanidin 3,5-diglucoside	1.3
2	cyanidin 3-glucoside	85.5
3	cyanidin 3-rutinoside	1.8
4	pelargonidin 3-glucoside	2.3
5	peonidin 3-glucoside	7.2
6	peonidin 3-rutinoside	1.9

Figure S1. HPLC profiles of phenolic components in *Lonicera caerulea* L. berry polyphenols (LCBP). LCBP was analyzed by using a HPLC system as described in the previous study [3]. (**A**) Profile of flavonoids and phenolic acids in LCBP. HPLC was performed at 280 nm. (-)-epicatechin (EC) and cyanidin 3-glucoside (C3G) accounted for 25.5% and 59.5% in the phenolic fraction, respectively. (**B**) Profile of anthocyanins in LCBP. Anthocyanins were detected at 520 nm, and six kinds of anthocyanin were identified with known standards. The diagrams represent the typical pattern of three HPLC profiles. The x and y axis represent retention time and intensity respectively.

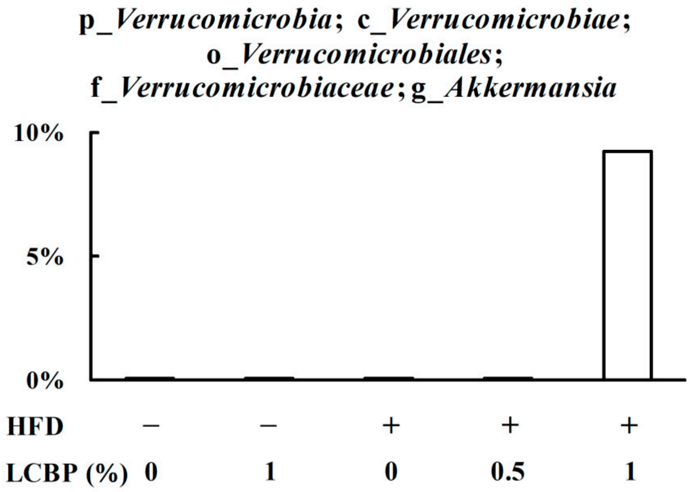

Figure S2. The effect of LCBP on the relative abundance of *Akkermansia*. Fecal microbiota were characterized by 16S rRNA gene sequencing, and the data represents the relative abundance of *Akkermansia*. p_, c_, o_, f_, and g_ represent phylum, class, order, family, and genus, respectively. HFD, high fat diet; LCBP, *Lonicera caerulea* L. berry polyphenols.
